# Influence of Selected Product and Process Parameters on Microstructure, Rheological, and Textural Properties of 3D Printed Cookies

**DOI:** 10.3390/foods9070907

**Published:** 2020-07-10

**Authors:** Cinu Varghese, John Wolodko, Lingyun Chen, Michael Doschak, Prem Prakash Srivastav, M. S. Roopesh

**Affiliations:** 1 Rural Development Centre, Indian Institute of Technology Kharagpur, Kharagpur 721302, India; cinuvarghese@iitkgp.ac.in (C.V.); pps@agfe.iitkgp.ac.in (P.P.S.); 2 Department of Agricultural, Food and Nutritional Science, University of Alberta, Edmonton, AB T6G 2P5, Canada; jwolodko@ualberta.ca (J.W.); lingyun.chen@ualberta.ca (L.C.); 3 Faculty of Pharmacy and Pharmaceutical science, University of Alberta, Edmonton, AB T6G 2P5, Canada; mdoschak@ualberta.ca

**Keywords:** 3D-printing, fill density, cookie dough, micro-CT, rheology, hardness

## Abstract

One of the major advantages of 3D food printing is the customizability in terms of structure, design, and nutritional content. However, printability of the ingredients and the quality of the 3D printed food products are dependent on several product and printing parameters. In this study, nutrient dense cookies were developed with underutilized ingredients including jackfruit seed powder and finger millet powder as base materials using 3D food printing. The hardness, rheological behavior, and microstructure of 3D printed cookies with different products (e.g., water butter ratio) and printing (e.g., fill density and temperature) parameters were analyzed. The 3D printed cookies were developed by extruding at 27 and 30 °C with fill density values of 50%, 70%, 90%, and 100% and water butter ratios of 3:10 and 6:5. The 3D-printed cookie dough exhibited a more elastic behavior with higher storage modulus values than the loss modulus. The hardness of the baked cookies was influenced by printing temperature, fill density, and water butter ratio of 3D printed cookie dough and their interactions. The closed porosity of 3D printed cookies increased while the open porosity decreased with an increase in fill density. The baking times required were longer for 3D-printed cookies with higher fill density values. Overall, this study shows the importance of considering the specific ingredient and printing parameters to develop high quality 3D-printed cookies.

## 1. Introduction

3D food printing (3DFP) is growing as a novel technology that can modify food to personal fondness, mostly in shape and hardness. Children and adolescents have increased nutritional requirements demanding a diet rich in protein, vitamins, calcium, iodine, phosphorus, and iron. Digitalization and customization of nutrients are some of the major advantages of 3DFP. The 3DFP has the potential to develop low cost, customized products for undernourished children and adolescents [1,2]. 3D food printers are modified 3D printers with unique pressurized mechanisms to extrude the raw material, i.e., mix of food ingredients in different forms such as powder, paste, or semi-solid. A variety of products can be made using these 3D food printers, namely customized chocolate, fresh pancakes, cookies, pizzas, and sugar candy [2,3]. The 3DFP has the advantage to develop texturized and palatable food shapes, which provides enjoyment in eating rather than the monotony of consuming pureed food such as mashed carrots, potatoes, and spinach [3]. Thus, in 3DFP, the most important parameter for texture variation in printed foods is the internal structure, which is dependent on the fill pattern and the fill density during printing. The fill pattern refers to the type of configuration that will form as a pattern within the printed construction. Fill density (F.D.) is the density of the structure (layer-to-layer gap) of the material upon deposition. The fill density values, including pattern and percentage, can be selected by the Cura software by slicing application used for 3DFP before the printing process [4]. Depending on the selection of fill pattern and the fill density, unique internal microstructure within the printed food can be developed.

One of the challenges in 3DFP is the printability of customized products. Printability is dependent not only on the physicochemical properties of the ingredients, but also the process parameters such as nozzle moving speed, nozzle height, nozzle diameter, extrusion rate, layer height, and printing temperature [5]. The printing parameters such as infill pattern and fill density also affect the stability of customized 3D-printed foods. Limited research was conducted on dimensional stability, which is also related to printability, after post-processing of 3D-printed foods. Pulatsu, Su, and Lin et al. [6] reported a modified formulation to create stable 3D shapes using the traditional recipe without adding any stabilizers. The authors highlighted the effects of sucrose, butter, and milk level on the extrudability and 3D-printability of the cookie dough and concluded that the formulation with low sugar level yielded structurally stable cookies after baking.

3D food printing allows better management of composition, taste, structure, and texture of foods and preparation of novel shapes, which offers novel product ideas in the food processing industry [7,8]. Gholamipour-Shirazi et al. pointed out that one of the advantages of 3D food printing was food personalization and customization of nutritional profiles for malnourished target groups [9]. Developing 3D-printed fortified snack foods such as fortified cookies is one of the main ways to deliver essential nutrients to children and adolescents. Moreover, 3D food printing could be used to improve visual aspects by developing foods that are highly appealing to consumers such as children and adolescents [7]. In this study, the cookies were developed with the use of ingredients such as finger millet flour and jackfruit seed flour. Finger millet flour is rich in calcium and dietary fiber [10] as well as jackfruit seed flour, which is gluten-free and rich in carbohydrates [11]. In addition, the cookies were fortified with vitamins and minerals, which satisfy the requirements of undernourished adolescents. However, printability and stability of gluten-free cookie dough is a challenge, which was not studied before with regard to 3D-printed foods.

It is important to understand the ingredient and dough properties, which influence the final product microstructure and texture of 3D-printed cookies. The influence of important parameters, including water butter ratio in cookie dough and printing temperature on the quality of printed cookies, was not studied extensively. During traditional baking, a lack of gluten and high amount of fiber in the ingredients resulted in a poor texture, irregular shape, and thickness of the cookies [12]. In this study, we hypothesized that standardized layering during 3D-printing can result in a uniform texture and microstructure during baking, which leads to high quality cookies. The main objective of this study was to assess the influence of ingredient properties and the process parameters on the development of high energy, gluten free, and nutrient-dense 3D-printed cookies. This study focused on understanding the influence of printing temperature, fill density, and the water butter ratio on the textural, rheological, and microscopic structure of the cookies.

## 2. Materials and Methods

### 2.1. Raw Materials

Jackfruit seed, finger millet, unsalted butter, granulated sugar, xanthan gum (Purix^TM^ Bakersville India Pvt. Ltd. Indore, Madhya Pradesh, India)*,* baking powder (Weikfoods private limited Pune, Maharashtra, India), soy protein isolate (Purix^TM^ Bakersville India Pvt. Ltd. Indore, Madhya Pradesh, India), and vitamin and mineral premix (Hexagon Nutrition Pvt. Ltd. Mumbai, India) were used as ingredients for 3D-printed cookies. The cookies were developed using underutilized ingredients such as jackfruit seed powder and finger millet powder as the base. The Jackfruit seeds were roasted, ground, and sieved using a 250-µm mesh sieve [13]. Finger millet was sprouted, roasted, and sieved through an 80-mesh sieve [14].

### 2.2. Dough Preparation

Two cookie dough formulations (A and B) were used to prepare 3D-printed cookies (Table 1). Butter was kept at room temperature (~23 °C) for 15 min to make it easy for blending. The powdered sugar and butter were blended until it gives a smooth texture. Jackfruit seed powder, finger millet powder, xanthan gum, baking powder, and vitamin and mineral premix were mixed uniformly and then blended with the butter-sugar mixture. After blending water was added, the mixture became a type of dough paste.

### 2.3. 3D Food Printing

A FoodBot 3D-printer (Shyin Tech., Hangzhou, China) based on the principle of micro-extrusion was used to prepare 3D-printed cookies. The 3D-printer consisted of an operating system and a control screen. Using the control screen, users are able to connect the printer to Wi-Fi, choose the printing model, adjust the printing speed, select the printing temperature, move the position of barrel, and extrude the food material.

**Figure 1:**
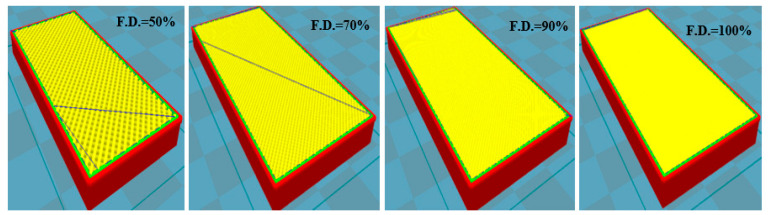
Representative images of 3D designs developed for cookies with different fill densities (F.D.).

**Figure 2:**
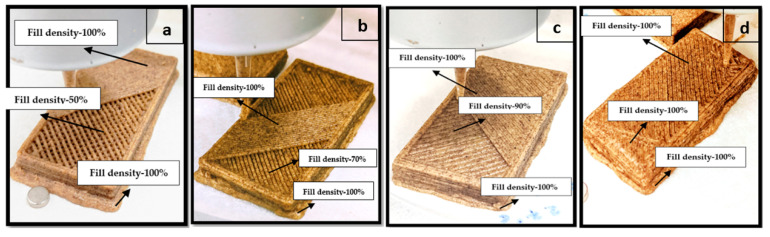
Representative images of 3D-printed dough structure with different fill density values of (**a**) 50%, (**b**) 70%, (**c**) 90%, and (**d**) 100%.

The length, width, and thickness of the 3D design developed for the cookies in Tinkercad program were 6, 3, and 0.7 cm, respectively. The 3D model in .stl file was converted to a G-code, and the specific parameters were selected using CURA software. The layer thickness of each layer was determined to be 0.20 mm, the nozzle size was measured to be 0.5 mm, and the top and bottom layer thickness was set to 0.6 mm based on preliminary trials. The 3D design for the printing of cookies is presented in Figure 1. The top and bottom layers were filled as solid layers with 100% fill density to obtain a stable, strong base, and more finishing was completed at the top in every cookie sample (Figure 2). The heater attached to the barrel was able to heat the syringe and the material inside. The printer nozzle diameter and print speed used in the study were 1.55 mm and 50 mm/s, respectively. The cookie dough was filled inside a syringe and placed inside the barrel connected to the 3D-printer. The influence of different product and printing parameters such as fill density, temperature, and water butter ratio on the hardness and microstructure of 3D-printed cookies were evaluated. The temperature of the barrel (printing temperature) connected to the printer was selected as 27 and 30 °C. The syringe, in which the product was filled, was placed inside the barrel during printing. Hence, the product inside the syringe was assumed to achieve the barrel’s temperature in a short period of time. The fill density values of 50%, 70%, 90%, and 100% and the water butter ratios of 3:10 and 6:5 based on weight were used as the main parameters in this study. The dough was extruded from the syringe through an extrusion head and printed on a platform held by the pedestal. The 3D-printed cookies were baked at 150 °C using an oven. The weights of the cookies printed at different fill densities were different. The baking time was determined by monitoring the moisture content from weight data in every 1 min until the target moisture content (dry basis) reached ~4%.

### 2.4. Rheological Analysis

Rheological parameters (storage and loss modulus values) of the 3D-printed porous dough structure with different water butter ratios, i.e., (a) 3:10 and (b) 6:5 printed at 27 °C and 30 °C before baking were measured using a rheometer (TA instruments Trios, New castle, PA, USA). The rheometer was connected to a parallel plate with a diameter of 40 mm and a gap of 20 mm between the plate and the printed dough sample. For rheological analysis, dough with different formulations were printed first with dimensions of a 45-mm diameter and a 3-mm thickness. The 3D structure samples were placed between the parallel plates, and the edges were trimmed with a spatula. Dynamic viscoelastic properties were determined at an oscillatory frequency sweep mode. The angular velocity varied from 1 to 100 rad/s, and all the measurements were performed within the identified linear viscoelastic region at 0.1% strain. The storage modulus (G’) and loss modulus (G”) for the 3D structures were recorded [15]. Each rheological measurement was evaluated in triplicates.

### 2.5. Micro CT Analysis

The microstructure of the developed cookies was analyzed at different fill density values of 50%, 70%, 90%, and 100%. The printed cookies after baking were kept at room temperature for 2 h. The 3D images of the cookies were developed using a bench-top Micro-CT imager (SkyScan 1076, Bruker-MicroCT, Kontich, Belgium) at 18-µm voxel image resolution with 70 kV, 100 µA, and a 1-mm aluminum filter to remove low energy photons. Raw image projections of the cookies were reconstructed using a modified Feldkamp back-projection algorithm available with the bundled vendor software (Nrecon 1.6.1.5, Bruker-MicroCT, Kontich, Belgium). Representative region of interest (ROI) were sampled from the cross-sectional reconstructed images for all cookies and analyzed for structural parameters using vendor bundled CT-Analyze software (ver 1.11.6.0, Bruker-MicroCT, Kontich, Belgium). The same minimum and maximum threshold values were used to binarize each cookie in order to facilitate image analysis [16,17]. The structural parameters determined using MicroCT analysis were open porosity, closed porosity, and total porosity. The parameters were calculated from three-dimensional analysis of each volume of interest (VOI) stack of binarized slices through the entire thickness of each cookie.

### 2.6. Texture Analysis

The Texture Analyzer (model TA-XT2i, Stable Microsystems, Surrey, UK) was used to measure the hardness (as fracture force) of cookies with a puncture probe that has a diameter of 4 mm, 35 mm long, a trigger force of 0.005 kg, and load cell of 50 kg. The textural studies were conducted at a pretest speed of 1 mm/s, test speed of 3.0 mm/s, post-test speed of 10 mm/s, and a distance of 5 mm. The gap between the two bottom supports was adjusted to 50 mm. The peak value of maximum force was recorded as hardness at a point when the baked cookies were broken into two major pieces [18]. This maximum force (g) at the breaking point characterized the breaking strength of the cookie. Mean value in triplicates were reported as the maximum force.

### 2.7. Data Analysis

Data on the response variables, that is, complex viscosity, hardness, and moisture content, were analyzed with SAS 9.4 (SAS Institute, Cary, NC, USA) using the two-way Analysis of Variance (ANOVA) analysis. The microscopic structure parameters were analyzed with IBM SPSS Statistics for Windows, Version 22.0. (IBM Corp., Armonk, NY, USA). Significant difference between the means was determined using Duncan’s multiple range test (DMRT). A value of *p* < 0.05 was selected as statistically significant.

## 3. Results and Discussion

### 3.1. Effect of Fill Density, Temperature, and Water Butter Ratio on the Printed Cookie Dough Rheology

Rheological properties of a formulation define the flow behavior and the elasticity of the product. It could be an index to know the printability of the formulation. Figure 2 represent the images of the 3D-printed porous dough structures with different fill densities. For gluten-free formulation as used in this study, modifications are required to achieve suitable printability of cookie dough. The addition of hydrophilic colloids and selection of an appropriate water butter ratio in cookie dough could help achieve better printability. Kim et al. [19] reported that cookie dough added with 0.5% xanthan gum showed a defined shape and smooth printing. In 3D food printing, extrusion and printing are easier if the elastic and viscous properties of dough balanced each other [20].

Storage (G’) and loss modulus (G”) are important rheological properties. Storage modulus is the elastic response of the material or elastic modulus. The G’ is related to the structural stability of the printed structure to form a gel-like elastic product with strength and adhesiveness.

**Figure 3:**
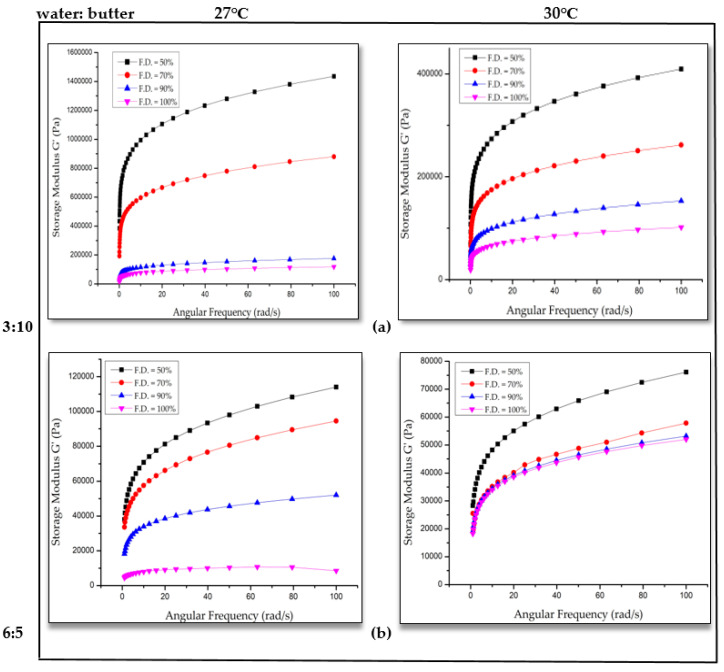
Storage modulus (G’) versus angular frequency of dough at different water ratios (**a**) 3:10 and (**b**) 6:5 at 27 °C and 30 °C.

When the applied force is less than the intermolecular force, the material behaves like an elastic solid but not the ideal one [21]. With an increase in the fill density (F.D.), a decrease in storage modulus of printed dough structure was observed (Figure 3). That means, a 3D structure with 50% fill density showed higher elastic nature than the 3D structure with 100% fill density. The exact reason for the decrease in storage modulus with increase in fill density of the 3D structure is clear as the intermolecular forces could be higher with an increase in fill density. The deposition of more dough and decrease in the infill space with an increase in fill density led to a denser structure. In addition, cookie dough printed at 30 °C with a water butter ratio of 6:5 showed less of a difference in storage modulus values for 90% and 100% fill density printed cookie dough [22].

With an increase in printing temperature from 27 to 30 °C, decrease in values of storage modulus was observed (Figure 3a,b). It is important to note that 27 °C and 30 °C were the printing barrel temperatures. Water and butter play an important role in the viscoelastic nature of the 3D-printed porous dough structure. The increase in water content and decrease in butter content resulted in a combined effect to the decrease in G’ and G” values. The melting point of butter is ~32 °C. There may be a difference in structural reorganizations after printing at different temperatures, which might have contributed to decrease in G’ values. With increased temperature, the 3D-printed structure showed lower strength and rigidity. Increase in temperature can cause melting of fat in the dough structure and, thus, storage modulus is decreased [20]. With an increase in butter content (water:butter = 3:10), the G’ values were increased, which was more pronounced for printing temperature of 30 °C. Printed cookie dough structure with 3:10 water butter ratio at 27 °C exhibited the most defined and intact shape. The addition of butter improves the stability of the microstructure by reconstructing the pores in the internal structure and forming stiff dough networks with high gel strength and, at the same time, it decreases the moisture content and improves the rigidity of pore walls. However, the addition of large quantities of butter may damage the internal structure [23].

The easiness in printing and extrusion is possible with the control of temperature in the case of 3D printing. The dough used in this study had a high apparent viscosity. Therefore, a minimum amount of heat was required for extrusion [24]. In this study, the minimum temperature of 27 °C was required to extrude the dough from the printer barrel smoothly. However, lower temperature gives more defined printing and consistency of the dough.

**Figure 4:**
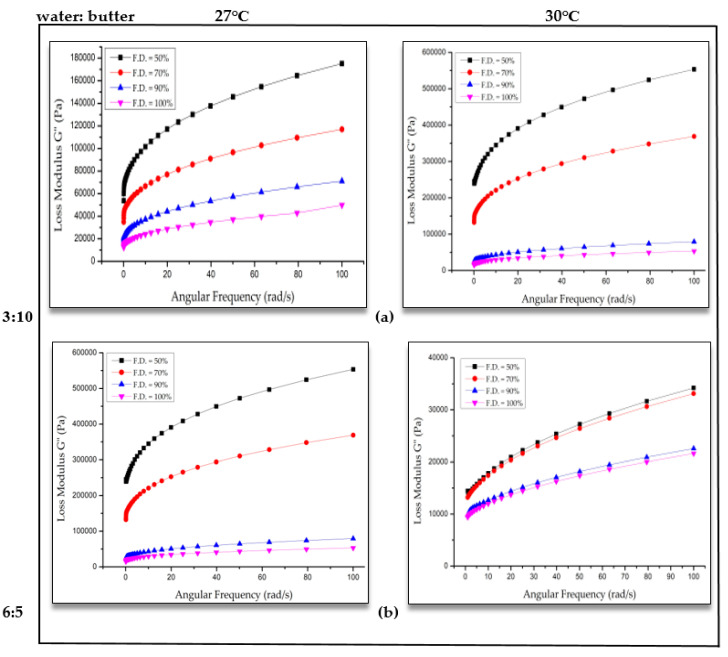
Loss modulus (G’’) versus angular frequency of dough at different water ratios: (**a**) 3:10 and (**b**) 6:5 at 27 °C and 30 °C.

Loss modulus (G”) is the measure of the viscous nature of the material, which is related to the flow behavior of the cookie dough through the extruder nozzle of the 3D printer. When the applied force is higher than the internal forces between the particles, the material collapses and the mechanical energy is released. Then, the material begins to flow. With an increase in fill density, there was a decrease in loss modulus, which is similar to the storage modulus (Figure 4). However, the exact reason for this observation is not clear. Increase in fill density resulted in an increase in closed pore volume and closed porosity of the 3D-printed cookies after baking were observed (discussed in the next section). This could be related to the decrease in storage and loss modulus values of the 3D dough structures. This closed porosity information was determined for baked cookies and not for the 3D-printed dough structures. For 30 °C printing temperature, the G” values were similar for 3D-printed porous dough structures with 90% and 100% fill density (Figure 4). The storage modulus was higher than the loss modulus, which showed that the printed dough structures exhibited a more elastic behavior than the viscous behavior and the ability to form an elastic gel-like structure.

**Figure 5:**
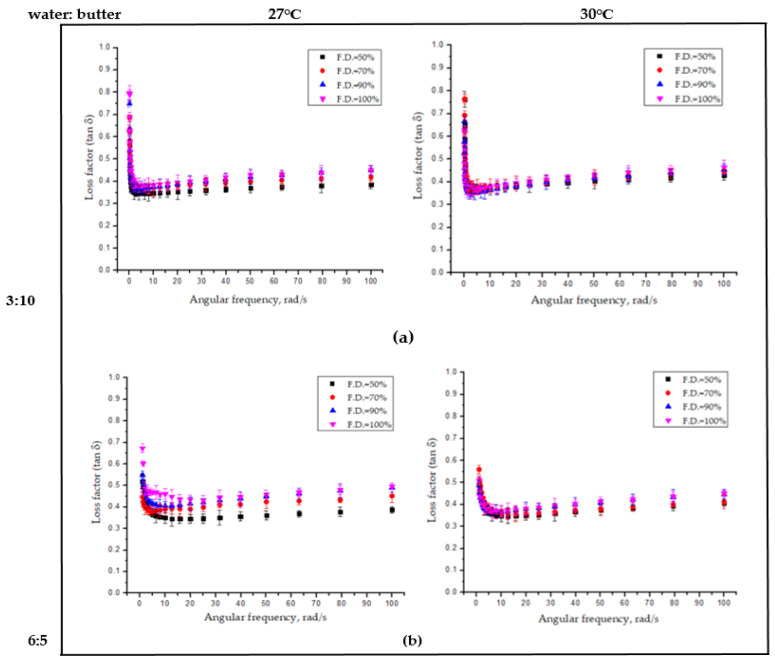
Loss factor (tanδ) versus frequency of 3D-printed dough structure at different water ratios: (**a**) 3:10 and (**b**) 6:5 at 27 °C and 30 °C, respectively.

The loss factor (tanδ=G”/G’) is a measure of internal friction of a material, which represent the ratio of the loss modulus and storage modulus. A higher value of the loss factor (tanδ > 1) corresponds with a viscous nature of material and vice versa [25]. In this study, the change in dynamic viscoelastic properties of the 3D-printed dough structure was greatly influenced by the combined effect of the water butter ratio, temperature, and fill density (Figure 5). The tanδ values were less than 1, which showed that the 3D porous dough structures were more solid-like and elastic than liquid-like. For the porous structure with 100% fill density, the tanδ values were generally greater than those with 50% fill density, especially at higher angular frequency levels, which indicates their more viscous nature than elastic nature with an increase in fill density. The effect of the water butter ratio on the visco-elastic properties was interpreted by the water acting as a plasticizer and as an inert filler, which change the visco-elastic properties proportionally and butter as lubricant-enhancing [26]. The increase in temperature cause the butter to melt (melting point of butter is ~32 °C) and, thus, enhances the extrusion and the elastic nature of the printed dough.

**Figure 6:**
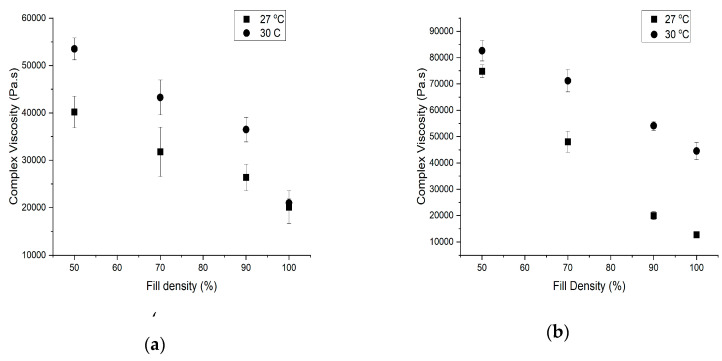
Complex viscosity values at different fill densities, temperatures, and (**a**) water butter ratios: 3:10 and (**b**) water butter ratio of 6:5. Values are mean ± standard deviation (*n* = 3).

Complex viscosity of 3D-printed porous dough structures increased with an increase in printing temperature and decreased with an increase in fill density (Figure 6) [27]. We found a significant interaction (*p* < 0.05) between the temperature and fill density on the complex viscosity of the 3D structure with both water butter ratios. The cookie dough printed with 6:5 water butter ratio at 27 °C and 30 °C showed higher differences in the complex viscosity [28]. The complex viscosity values changed with butter content, depending on the fill density of the 3D dough structure (Figure 6). Increase in G’ and G” with the addition of butter resulted in more stiffness and a defined shape in the internal microstructure and, hence, an increase in the complex viscosity. At constant strain, the storage modulus has a higher value than the loss modulus, which represents the nature of a typical elasto-viscous stiff body [29]. The cookies printed with a 3:10 water butter ratio showed less of a difference in complex viscosity at 27 °C and 30 °C. In the case of a water butter ratio of 6:5, the addition of more water caused more of a reaction with xanthan gum, which could have resulted in a more viscous dough and an increase in complex viscosity. The effect of temperature on the complex viscosity of 3D porous dough structure was a combined effect of fill density, addition of butter, and decrease in water content [30].

### 3.2. Effect of Fill Density on the Microscopic Structure of Cookies

3D food printing is a powerful technology that has not yet explored the influence of printing parameters, such as the fill density on the microscopic structure. Several bakery products are porous. Therefore, the knowledge about the microscopic structure gives insight about the mechanical and textural differences in different bakery recipes [31]. Micro-CT technology has previously been shown to be a useful method to study the porous network and the porosity of freeze died salmon and their relation to phase transitions during frozen storage [32]. In this study, micro CT technology was used to non-invasively measure the effect of different fill density on the porous network inside the cookies with a water butter ratio of 3:10, printed at 27 °C.

**Figure 7:**
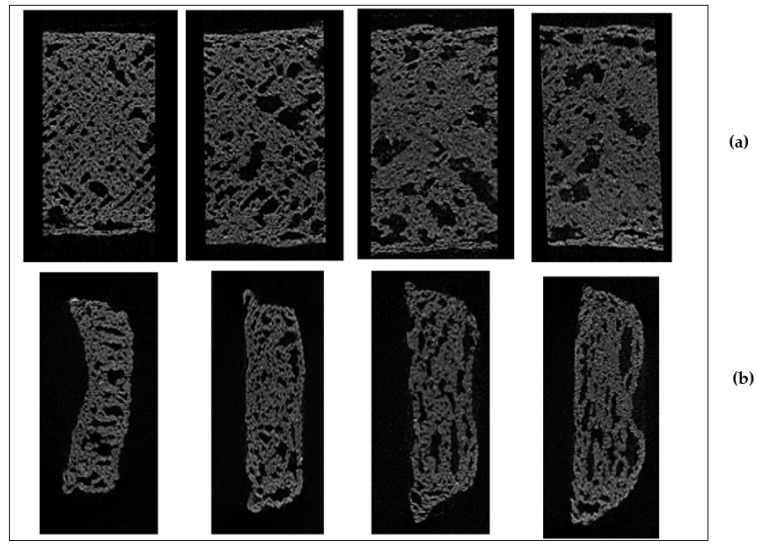
Representative images of (**a**) top view of reconstructed cross-sectional Micro CT images of baked cookies with 50%, 70%, 90%, and 100% fill density. (**b**) Cross-sectional view of reconstructed cross-sectional Micro CT images of baked cookies with 50%, 70%, 90%, and 100% fill density.

The printing patterns with different fill densities are presented in Figure 7a,b. Observations revealed that, with increased fill density, the 3D-printed cookies became more robust in the structure. However, rheological data showed a decrease in elastic modulus and stiffness of dough network of printed cookie dough. Figure 7a,b show the reconstructed top view and side view of cross-sectional micro CT images of the cookies. Image analysis was able to distinguish the bubbles (closed pores) and the connecting network (open pores) [33]. Increased puffiness was observed in cookies printed with 90% and 100% fill density as presented by the side view of cross-sectional micro CT images of the baked cookies (Figure 7b). The addition of baking powder triggered a chemical reaction when it was moistened with the dough and produced carbon dioxide (CO_2_), which could result in the expansion of cookies [34]. Therefore, cookies with 100% fill density exhibited a closed structure. The escape of CO_2_ was probably less in cookies with 90% and 100% fill density values when compared to other fill densities, which resulted in a puffy texture.

Numerical physical characteristics such as percentage object volume, open porosity, closed porosity, and total porosity were determined from the micro CT data using 3D scan software (Table 2). It was clearly evident that fill density had an effect on closed and open porosity (Table 2). The open pore space was higher in 50% fill density cookies than the 100% fill density, as observed from the top view and front view of cross-sectional micro CT images (Figure 7a,b). For 100% fill density, a higher amount of dough was used to fill the structure, which likely decreased the open porosity. The 3D data also shows that 50% fill density indicates more connectivity between the pores than the cookies with other fill densities. The closed porosity and volume of closed pores showed an increasing pattern with an increase in fill density from 50% to 100% but, on the other hand, open porosity and volume of open pores decreased with an increase in the fill density. Additionally, total volume of pore space and total porosity decreased with an increase in fill density (Table 2).

### 3.3. Effect of Fill Density, Temperature, and Water Butter Ratio on Hardness

**Figure 8:**
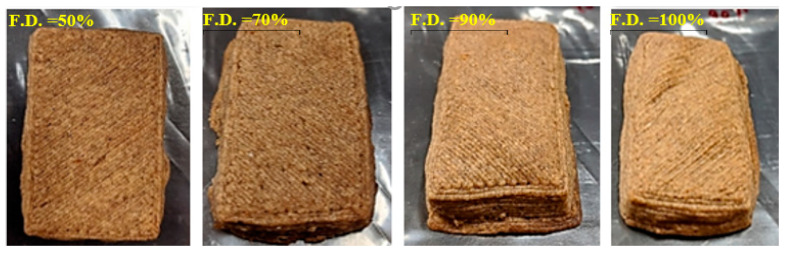
Representative images of cookies with different fill densities after baking.

**Figure 9:**
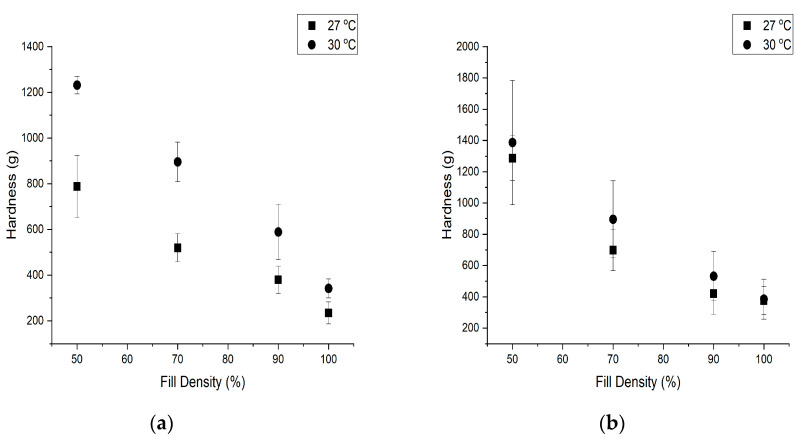
Effect of fill density, temperature, and (**a**) water butter ratio of 3:10. (**b**) Water butter ratio of 6:5 on hardness of baked cookies. Values are mean ± standard deviation (*n* = 3).

The 3D-printed cookies with different fill densities exhibited different hardness after baking. The representative images of 3D-printed cookies after baking are given in Figure 8.The change in the hardness of the baked cookies with different fill density, temperature, and water butter ratio were studied and presented in Figure 9. For 3D-printed cookies with water butter ratio of 3:10, a significant interaction (*p* < 0.05) between temperature and fill density on hardness of the cookies was observed. This means that, for the 3D-printed cookies with a water butter ratio of 3:10, the effect of temperature on hardness of the cookies was dependent on the fill density and vice versa.

Cookies baked with 100% fill density observed more puffiness and expansion than the cookies with a fill density of 50%. For a 3D-printed cookie with 100% fill density, the higher closed porosity and closed pore volume could have influenced its hardness and structure as compared to open porosity and open pore volume. More rise in cookies with 100% fill density was likely due to the increased closed porosity. The less opportunity of CO_2_ to escape in the 100% fill density cookies compared to those with 50% fill density likely resulted in the puffiness of cookies.

Shape stability of all cookies was maintained after printing and baking. The moisture content of cookies with 50% fill density decreased at a faster rate when compared to cookies with other fill densities. The larger contact surface area of the 50% fill density cookies might have accelerated the baking process. Zhang et al. observed that the “honeycomb” structure showed a more brittle nature with increased hardness and less stickiness when compared to the concentric structure [15]. The effect of fill density was likely primarily on the baking and then on hardness.

Water acts as a plasticizer and butter acts as a lubricant, helping the cookie dough to print quickly and smoothly [6]. To gain the printable consistency, water was added with butter with ratios of 3:10 and 6:5. 

For 3D-printed cookies with a water butter ratio of 6:5, no significant interaction (*p* ≥ 0.05) between temperature and fill density on hardness of the cookies was observed. With an increase in printer temperature, hardness of the cookies showed an increase for cookies with a water butter ratio of 3:10 (Figure 9). The increased temperature resulted in less viscous dough due to the melting of the butter and likely lower pore volume. Hence, higher hardness was achieved after printing [6]. In addition, the flow ability of the mixture through the nozzle during printing was enhanced at 30 °C, which helped produce more defined printing.

### 3.4. Effect of Fill Density on the Time of Baking

**Figure 10:**
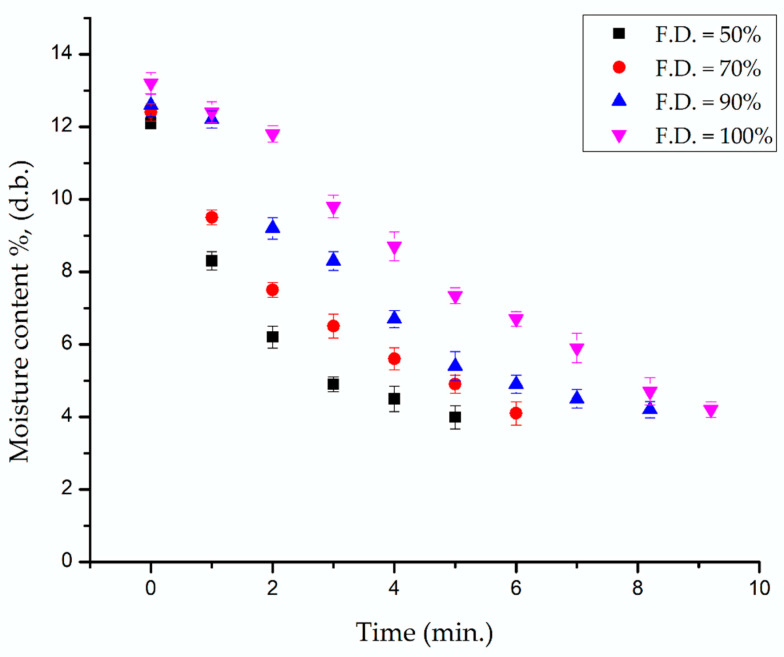
Baking times for 3D-printed cookies with different fill density values with a water butter ratio of 3:10 printed at 27 °C.

The initial moisture content of the printed cookies was 12–13% (dry basis). In the current study, moisture contents were determined by measuring the weight in every 1-min interval until the moisture content reached ~4% (dry basis). The weight of the 3D-printed cookies was dependent on the fill density. Amount of dough used to fill the inner part of samples was higher with increased fill density. Vapour produced during baking likely increased the size of the pre-existing pores and also created new ones. A similar study was reported by C. Severini et al. When the greater infill was used, more dough was extruded and a greater number of new pores were created during cooking due to vapour [7]. The drying time required to achieve a certain weight or final moisture was also different, which depends on the fill density of the printed cookies during baking. The baking times for printed cookies with 50%, 70%, 90%, and 100% fill density with a water butter ratio of 3:10, printed at 27 °C, are presented in Figure 10. Statistical analysis of the moisture content of 3D-printed cookies with the selected fill density values, considering the first five baking times (1, 2, 3, 4, and 5 min) showed a significant interaction between baking time and fill density on the moisture content of 3D-printed cookies. This means that the effect of baking time on moisture content depended on fill density and vice versa. Longer baking times (to achieve the target of ~4% moisture content) were required for printed cookies with higher fill density values. The average final moisture contents of the cookies after baking ranged between 3.9% to 4.7%, depending on the fill density values. This can be attributed to the change in heat and mass (moisture) transfer properties when the fill density values and weights are changed. The total porosity values of baked cookies were smaller for cookies with higher fill density (Table 2), which shows less opportunity of moisture to transfer through the cookies during baking. This affects the baking time. In addition, the moisture content decreased at a faster rate in 50% and 70% fill density baked cookies compared to other cookies because of its larger contact surface area. Additionally, the lower visco-elastic properties of the printed dough with higher fill density could also influence the molecular interactions of water with other components in the cookies, which affects their removal rate during baking.

However, this argument needs further research for justification. The final physical properties of the printed cookies were dependent on the printing parameters as well as the formulation. Since the printability of cookie dough and quality of the final product are dependent on several products and printing parameters, future optimization is required in order to improve printability and develop high-quality 3D-printed foods.

## 4. Conclusions

In this study, the influence of different printing and product parameters on the rheological properties of 3D-printed cookie dough structure and microstructure and hardness of baked 3D-printed cookies were determined. The storage modulus of the cookies was higher than the loss modulus, which points out that the cookies showed more elastic behavior than the viscous behavior. Both storage modulus and loss modulus values of 3D porous dough structure decreased with an increase in fill density. Higher closed porosity values in cookies were observed when the fill density values were increased during 3D printing, while the open porosity decreased with an increase in the fill density. Cookies baked with 100% fill density exhibited higher softness than the cookies with a fill density of 50%. Longer baking times were required for cookies with higher fill density values, which means that the microstructure of the 3D-printed cookies had a great influence on the moisture transfer rate during baking. Results indicated that cookies with 90% fill density and water butter ratio of 3:10, extruded at 27 °C, showed better-defined printing and outcomes. Future optimization of the ingredient and printing parameters may be required to develop high quality 3D food printed products.

## Figures and Tables

**Table 1:** Formulation of dough considered in this study.

Ingredients	A^1^	B^2^
Jackfruit seed flour (%)	23.19	23.99
Finger millet flour (%)	21.55	22.28
Vitamin and mineral premix (%)	1.41	1.46
Soy protein isolate (%)	14.08	14.56
Butter (%)	16.57	8.56
Water (%)	4.97	10.29
Xanthan gum (%)	0.83	0.86
Baking powder (%)	0.83	0.86
Sugar (%)	16.57	17.14

A^1^: Formulation with water butter ratio of 3:10 (g). B^2^: Formulation with water butter ratio 6:5 (g).

**Table 2:** 3D analysis of binary images performed by CTAn scanning software.

Parameters	Fill Density			
	50%	70%	90%	100%
Volume of Closed Pores (mm)^3^	2.55 ± 0.20 ^a^	3.87 ± 0.75 ^a^	5.79 ± 0.78 ^b^	7.69 ± 1.01 ^c^
Closed Porosity (%)	0.62 ± 0.06 ^a^	0.73 ± 0.10 ^ab^	0.91 ± 0.15 ^b^	1.20 ± 0.20 ^c^
Volume of Open Pore Space (mm)^3^	837.6 ± 2.1 ^d^	465.7 ± 34.6 ^c^	398.3 ± 11.5 ^b^	267.3 ± 17.8 ^a^
Open Porosity (%)	67.0 ± 4.0 ^a^	46.6 ± 10 ^b^	28.4 ± 1.0 ^c^	21.1 ± 1.2 ^c^
Total Volume of Pore Space (mm)^3^	868.0 ± 4.1 ^d^	469.6 ± 35.0 ^c^	404.1 ± 11.4 ^b^	275.0 ± 5.3 ^a^
Total Porosity (%)	67.6 ± 4.1 ^c^	50.6 ± 5.8 ^b^	29.3 ± 9.5 ^a^	22.3 ± 2.9 ^a^

Values are mean ± standard deviation (*n* = 3). Means with different superscripts within the same row are significantly different when using the Duncan multiple range test. (*p* < 0.05).
